# Rat astrocytes during anoxia: Secretome profile of cytokines and chemokines

**DOI:** 10.1002/brb3.1013

**Published:** 2018-06-04

**Authors:** Zeinab Adel Samy, Lulwa Al‐Abdullah, Marian Turcani, James Craik, Zoran Redzic

**Affiliations:** ^1^ Faculty of Medicine Department of Physiology Kuwait University Kuwait Kuwait; ^2^ Faculty of Medicine Department of Biochemistry Kuwait University Kuwait Kuwait

**Keywords:** astrocytes, cytokines, hypoxia/ischemia

## Abstract

**Introduction:**

The precise mechanisms of the inflammatory responses after cerebral ischemia in vivo are difficult to elucidate because of the complex nature of multiple series of interactions between cells and molecules. This study explored temporal patterns of secretion of 30 cytokines and chemokines from Sprague Dawley rat astrocytes in primary culture in order to elucidate signaling pathways that are triggered by astrocytes during anoxia.

**Methods:**

Primary cultures of rat brain astrocytes were incubated for periods of 2–24 hr in the absence of oxygen (anoxia) or under normal partial pressure of oxygen (controls). Simultaneous detection of 29 cytokines and chemokines in the samples was performed using a rat cytokine array panel, while the temporal pattern of angiopoietin‐1 (Ang‐1) secretion was determined separately using ELISA. Wilcoxon–Mann–Whitney test was used to compare normoxic and anoxic samples and the Hodge–Lehman estimator with exact 95% confidence intervals was computed to assess the size of differences in cytokine secretion. The obtained data were imported into the Core Analysis tool of Ingenuity Pathways Analysis software in order to relate changes in secretion of cytokines and chemokines from astrocytes during anoxia to potential molecular signal networks.

**Results:**

With the exception of Ang‐1, concentrations of all cytokines/chemokines in samples collected after anoxia exposure were either the same, or higher, than in control groups. No clear pattern of changes could be established for groups of cytokines with similar effects (i.e., pro‐ or anti‐inflammatory cytokines). The pattern of changes in cytokine secretion during anoxia was associated with the HIF‐1α‐mediated response, as well as cytokines IL‐1β and cathepsin S pathways, which are related to initiation of inflammation and antigen presentation, respectively, and to ciliary neurotrophic factor.

**Conclusions:**

These in vitro findings suggest that astrocytes may play a role in triggering inflammation during anoxia/ischemia of the brain.

## 1 SIGNIFICANCE STATEMENT

The precise mechanisms of inflammation in the brain after cerebral ischemia (CI) have been difficult to elucidate because of the complexity of sequences of interactions between cells and molecules. In this study, cultured rat astrocytes were exposed to anoxia for 2–24 hr and concentrations of 30 cytokines and chemokines in the cell culture medium samples were estimated. These data were analysed using the Core Analysis tool of Ingenuity Pathways Analysis software. This analysis revealed that anoxia can activate astrocytes and that astrocytes may play an important role in triggering inflammation and antigen presentation during CI.

## 2 INTRODUCTION

Functional coupling between neurons, astrocytes, vascular smooth muscle cells, pericytes, and brain endothelial cells, which compose a functional unit known as the neurovascular unit (NVU) (Iadecola, 2017), is mainly achieved via paracrine and autocrine signaling mediated by a repertoire of cytokines and chemokines. Astrocytes play a particularly important role in these communication processes by secreting a number of signaling molecules, known as cytokines and chemokines, which include transforming growth factor‐β (TGF‐β), glial‐derived neurotropic factor (GDNF) (Igarashi et al., 1999), fibroblast growth factor (FGF) (Igarashi et al., 1999), nerve growth factor (NGF), ciliary neurotrophic factor (CNTF) (Hu et al., 1997), brain derived neurotrophic factor (BDNF), vascular endothelial growth factor (VEGF), insulin‐like growth factor‐1(IGF‐1), leukemia inhibitory factor (LIF) (Farina, Aloisi, & Meinl, 2007). C‐X‐C motif ligand (CXCL) 1, CXCL3, CXCL6, and CXCL8 (Lu et al., 2005).

When oxygen supply to a particular area of the brain becomes inadequate, hypoxia may trigger the pathological pathways of an ischemic cascade, including necrotic cell death in the ischemic core (within minutes of the onset of hypoxia) and apoptotic cell death in the ischemic core and surrounding areas, processes referred to as ischemic stroke. In response to ischemia, the NVU produces and secretes cytokines, chemokines, cell adhesion molecules, and matrix metalloproteinases (Siniscalchi et al., 2014), which trigger inflammation,^1^ and degradation of basement membrane and of the tight junctions between endothelial cells (Dirnagl, 2012). These processes attract immune cells from the blood and permit their entry into the ischemic area of the brain, as well as in sites of secondary neurodegeneration (Jones et al., 2018), which enhances inflammation.

However, details of the cascade of signals and effector responses that are triggered by ischemia, and a sequence(s) of events that enhance inflammation in the brain following stroke, are not well‐understood. It is not clear to what extent secretion of cytokines by particular cell types of the NVU during ischemia contributes to the development of inflammation. A recent study has revealed that oxygen–glucose deprivation triggers activation of resting microglia in primary culture, but presence of other cell types was required to induce the proinflammatory phenotype in these cells that is seen in vivo after middle cerebral artery occlusion (Barakat and Redzic, 2015). Also, although It is known that astrocytes secrete several proinflammatory cytokines during cerebral ischemia in vivo, including IL‐1α, IL‐1β, IL‐6, TNF‐α MMPs, and IFN‐γ (Tuttolomondo, Di Raimondo, di Sciacca, Pinto, & Licata, 2008), it has also been observed that astrocytes challenged by oxygen–glucose deprivation released soluble factors that attenuated microglial inflammatory responses (Kim, Min, Seol, Jou, & Joe, 2010). Thus, it is not clear whether lowered partial pressure of oxygen alone, a condition that is not necessarily associated with marked ATP depletion if glucose is available, or ATP depletion in the absence of signaling from other cell types, can trigger secretion of cytokines and chemokines from astrocytes.

Inflammation is a complex series of interactions between inflammatory cells and molecules, so a clear understanding of the role that astrocytes play after the onset of cerebral ischemia cannot readily be established without exploring a time pattern of cytokines and chemokines that are released by these cells after the onset of hypoxia/ischemia.

We aimed this study to determine whether oxygen deprivation, in the absence of any other deleterious conditions or other cell types, would be sufficient to trigger a major change in secretion of cytokines/chemokines from astrocytes, and to ascertain whether the pattern of changes in the secretome profile under these conditions suggests a role of astrocytes in triggering inflammation, or not. We used recent advances in multiarray technology, which permit simultaneous detection of low concentrations of up to 29 cytokines and chemokines in small‐volume samples. Our findings suggest that astrocytes may play an important role in antigen presentation and in controlling inflammation during oxygen deprivation.

## 3 MATERIALS AND METHODS

Two to three days old Sprague Dawley (SD) pups of both sexes were used to produce primary cultures of astrocytes. Animals were obtained from the Animal Resource Center (ARC) in the Faculty of Medicine, Kuwait University. Pups were humanely killed by cervical dislocation. Animal care and handling protocols complied with the standards of the International Council of Laboratory Animal Sciences and with the guidance provided by the ARC. The protocol used on animals has been approved by ARC. All efforts were made to minimize the number of animals used when the study was designed.

### 3.1 Primary cultures

Primary cultures of astrocytes were produced and maintained as described earlier (Abbott, Dolman, Drndarski, & Fredriksson, 2012) and were further purified at 2 days after the seeding by 24 hr gentle shaking at 200 rpm at 37°C in CO_2_ independent cell culture medium (Invitrogen). The rationale behind this procedure was that after seeding astrocytes quickly attach to poly‐l‐lysine treated plastic, while oligodendrocytes, microglia, and other contaminating cells do not. Thus, shaking of the flasks at this stage improves purity of the astrocyte cultures. The same protocol was repeated at day 7 after the seeding. Cell culture medium was replaced every 2–3 days thereafter until cells reached >90% confluence; confluence was confirmed by phase contrast microscopy. Primary cultures were used for experiments ≈10 days after the seeding.

### 3.2 Immunocytochemistry

Primary cultures were fixed in 4% paraformaldehyde in phosphate buffered saline (PBS) for 15 min at room temperature. Cells were permeabilized by 15 min incubation in methanol at −15°C. Nonspecific binding of antibodies was prevented by 1 hr incubation in PBS that contained 10% fetal calf serum (FCS). Primary cultures were then incubated overnight at 4°C in PBS that contained 0.1% (v/v) Tween (PBST), 1% FCS, and rabbit polyclonal antibody to rat glial fibrillary acidic protein (GFAP) (Sigma‐Aldrich, catalogue number G9269, RRID: http://scicrunch.org/resolver/AB_477035) with either of the following two antibodies: mouse polyclonal antibody to rat platelet derived growth factor‐ beta receptor (Abcam catalogue number ab69506, RRID: http://scicrunch.org/resolver/AB_1269704) or mouse monoclonal antibody to rat alpha smooth muscle actin (Abcam catalogue number ab7817, RRID: http://scicrunch.org/resolver/AB_262054). All primary antibodies were used at 1:200 dilution. Goat polyclonal antibody to rabbit IgG conjugated to fluorescein isothiocyanate (FITC) (Abcam Catalogue number ab6717, RRID: http://scicrunch.org/resolver/AB_955238) and goat polyclonal antibody to mouse IgG conjugated to Cy‐5 (Abcam Catalogue number ab6563, RRID: http://scicrunch.org/resolver/AB_955068) were used as secondary antibodies. Nuclei were stained with 4′,6‐diamidino‐2‐phenylindole (DAPI). Cells were examined using fluorescence microscopy (Zeiss Axiovert 40CFL) at 100 ×  magnification. Images were acquired by AxioCam camera using Axio Vision 4.8 software.

### 3.3 Anoxia and control groups

Flasks with primary cultures were randomly selected to anoxia or to control groups.

Flasks in the anoxia group were transferred to a hypoxic glove‐box chamber (Plas BY Labs‐Lansing, MI, USA) with an atmosphere consisting of 5% CO_2_ and 5% H_2_ in N_2_. A palladium catalyst (Plas BY Labs‐Lansing) was used to remove any residual oxygen. Anaerobic conditions were confirmed with anaerobic strip indicators before and during the time course of incubation, following instructions by the manufacturer (Oxoid, Hampshire, UK). Taking into account the sensitivity of the indicator, partial pressure of oxygen was maintained below 0.3%. All buffers and media that were used for the anoxia experiments were placed in the glove‐box chamber for 24 hr prior to the experiments in order to equilibrate dissolved gases to the partial pressures of these gasses in the chamber. The temperature inside the chamber was maintained at 37 ± 1.5°C by an internally mounted heater.

Flasks in control group were incubated in atmosphere consisting of 5% CO_2_ in air at 37°C.

Before starting either of the two protocols, primary cultures were washed twice with PBS and then incubated in DMEM, supplemented with 10% FCS, antibiotic, antimycotic, and vitamin C. At the end of the incubation, cell culture medium was harvested and the cultures were subjected to cell viability analysis.

### 3.4 Cytokines and chemokines profiling

The harvested media were processed using Rat Cytokine Array panel A (R&D Systems, Minneapolis, USA), which can detect the following 29 cytokines and chemokines: Cytokine‐induced neutrophil chemoattractant‐1, 2 alpha/beta and 3; Ciliary neurotrophic factor; Fractalkine; Granulocyte‐macrophage colony‐stimulating factor; Soluble Intercellular Adhesion Molecule 1; Interferon gamma; Interleukins 1 alpha, 1 beta, 2, 3, 4, 6, 10, 13, and 17; Interleukin‐1 receptor antagonist and interferon gamma‐induced protein 10; C‐X‐C motif chemokine 5; Leukocyte‐endothelial cell adhesion molecule 1; Monokine induced by gamma interferon; Macrophage inflammatory protein‐1 alpha and 3 alpha; Regulated upon Activation, Normal T‐cell Expressed, and Secreted (RANTES); Chemokine (C‐C motif) ligand 17; TIMP metallopeptidase inhibitor 1; Tumor necrosis factor alpha and VEGF. Briefly, the array membranes were incubated with harvested media and antibody cocktail overnight on a rocking platform at 4°C. After several washings, membranes were briefly incubated with streptavidin conjugated to horseradish‐peroxidase (HRP). The membranes were washed several times and then exposed to chemoluminescent HRP substrate. Membranes were then placed in an autoradiography film cassette and exposed to X‐ray films for 10 min (Supporting Information Figure S1). The identity of a particular cytokine was determined by comparing array membranes with the map that was provided by the manufacturer (Supporting Information Figure S1). The intensity of the signal was quantified on the GS‐800 calibrated densitometer (Biorad), using Quantity One (1‐D Analysis software) (Bio‐Rad Laboratories, USA) and the concentration of each cytokine was obtained in the form of optical density expressed in square millimeters (mm^2^). Finally, the cytokine data were normalized to cell numbers in the flasks (mm^2^/10^6^ cells).

Since the array kit could not detect angiopoietin 1 (Ang1), which is an important cytokine in signaling in the NVU during hypoxia/anoxia (Sweeney, Ayyadurai, & Zlokovic, 2016), its concentrations in the samples was determined by a commercially available ELISA kit (Boster Biological Technology, USA). Absorbance at 450 nm was read in a microplate reader using the SoftMax Pro 5.2 software (Molecular Devices Corp., CA, USA) and the concentration of this cytokine (pg/ml) was determined from a standard curve.

### 3.5 Molecular network analysis

The experimental data, results of the statistical analysis and Entrez Gene IDs corresponding to cytokine and chemokine genes were imported into the Core Analysis tool of Ingenuity Pathways Analysis software (Qiagen, USA) in order to relate changes in secretion of cytokines and chemokines from astrocytes during anoxia and normoxia to potential molecular signal networks.

### 3.6 Statistical analysis

Four randomly selected flasks were used for every time point in each of the two experimental groups (anoxia and control), which produced four data samples for each group. No outliers were identified. Wilcoxon–Mann–Whitney test was used to compare the distribution of normoxic and anoxic sample data (StaXact, v. 7.0; Cytel Software, Cambridge, MA, USA). The test was applied only once on the same data set, thus no correction to avoid increase in the false discovery rate was needed. To assess the size of differences in cytokine secretion between normoxic and anoxic conditions, we computed the Hodge–Lehman estimator with exact 95% confidence intervals (StaXact, v. 7.0; Cytel Software). A *p*‐value <0.05 was considered statistically significant.

## 4 RESULTS

### 4.1 Primary cultures

**Figure 1:**
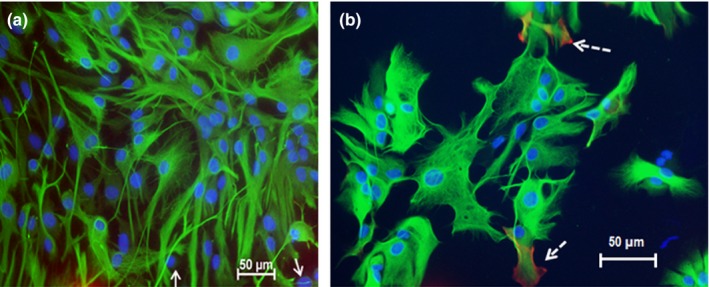
Purity of primary cultures of astrocytes. (a) Immunostaining of astrocytes in primary culture with FITC conjugated anti‐GFAP antibody and Cy‐5‐conjugated anti‐platelet derived growth factor‐beta antibodies. The vast majority of the cells were FITC fluorescence positive. A marginal Cy‐5 fluorescence at the bottom of the field that could not be clearly associated with any cell structure could be caused by nonspecific binding of antibodies. Very few cells were faintly stained with FITC (arrow); these could be fibroblasts. (b) Immunostaining of primary cultures with FITC conjugated anti‐GFAP antibody and cy‐5 conjugated anti‐smooth muscle actin antibody. A marginal cy‐5 fluorescence could be observed in some cells (arrows), though these cells are clearly positive for GFAP, so they are not VSMCs or pericytes. GFAP, glial fibrillary acidic protein

The vast majority of cells in primary cultures were clearly positive for GFAP only, although a few cells that did not stain for GFAP were also observed in almost every culture (Figure 1a, arrows). However, no cells were positive for PDGFR‐β, which is a marker of pericytes (Figure 1a), although in some cases red fluorescence that could be due to nonspecific binding of the secondary antibodies was observed. These imaging results indicated absence of pericytes from primary cultures used in these investigations. There was a marginal staining of a few GFAP‐positive cells with anti‐SMA antibodies (Figure 1b), which could also be due to unspecific binding of secondary antibodies, since no SMA‐positive cells (i.e., vascular smooth muscle cells and pericytes) express GFAP. Overall, the purity of the cultures, calculated as number of GFAP‐positive cells/number of nuclei in a visual field, exceeded 90%. Most of the nuclei that were not GFAP positive appeared to be pyknotic cells (Figure 1a). However, a minor contamination with other cell types, such as microglia and oligodendrocytes, could not be excluded.

### 4.2 Cytokine and chemokine secretion from rat brain astrocytes during normoxia and anoxia

**Figure 2:**
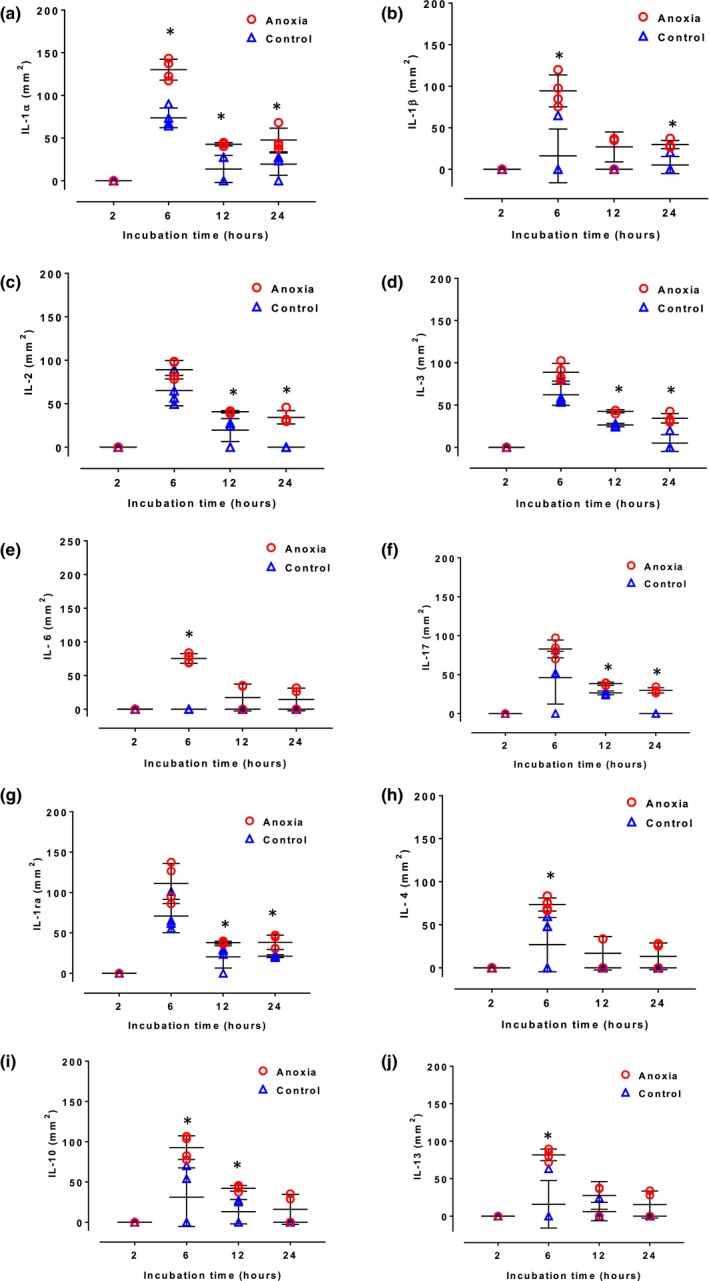
Secretion of interleukins by astrocytes at 2, 6, 12, and 24 hr of incubation in control conditions (triangles) and during anoxia (circles). Each symbol represents one sample. In some cases, data overlapped and hence it may appear that less than four samples are present for one‐time point. Long horizontal lines denote means, vertical lines denote standard deviations. Symbol * indicates significant difference between anoxia and control groups

IL‐1α, IL‐1β, IL‐2, IL‐3, IL‐6, and IL‐17 are pro‐inflammatory cytokines (Akdis et al., 2016), while IL‐1ra, IL‐4, IL‐10, and IL‐13 are anti‐inflammatory interleukins. No clear difference was found in the pattern of changes in concentrations of these cytokines after anoxia (between the pro‐ and anti‐inflammatory cytokines). After 2 hr anoxia, concentrations of these interleukins in the culture media harvested from primary cultures were almost the same as in the culture media harvested from control groups (*p *>* *0.05 for all) (Figure 2). After 6, 12, and 24 hr anoxia concentrations of most of these interleukins were higher than in the corresponding control groups, although differences were still marginal. The difference reached statistical significance for IL1a, 1b, 134, and 10 after 6 hr anoxia, for IL‐1a, 1ra, 2, 10, and 17 after 12 hr anoxia and mainly for proinflammatory cytokines IL‐1a, 1b, 2, 3, and 17 after 24 hr anoxia. Secretion of proinflammatory cytokine IL1a was consistently elevated after 6, 12, and 24 hr anoxia.

**Figure 3:**
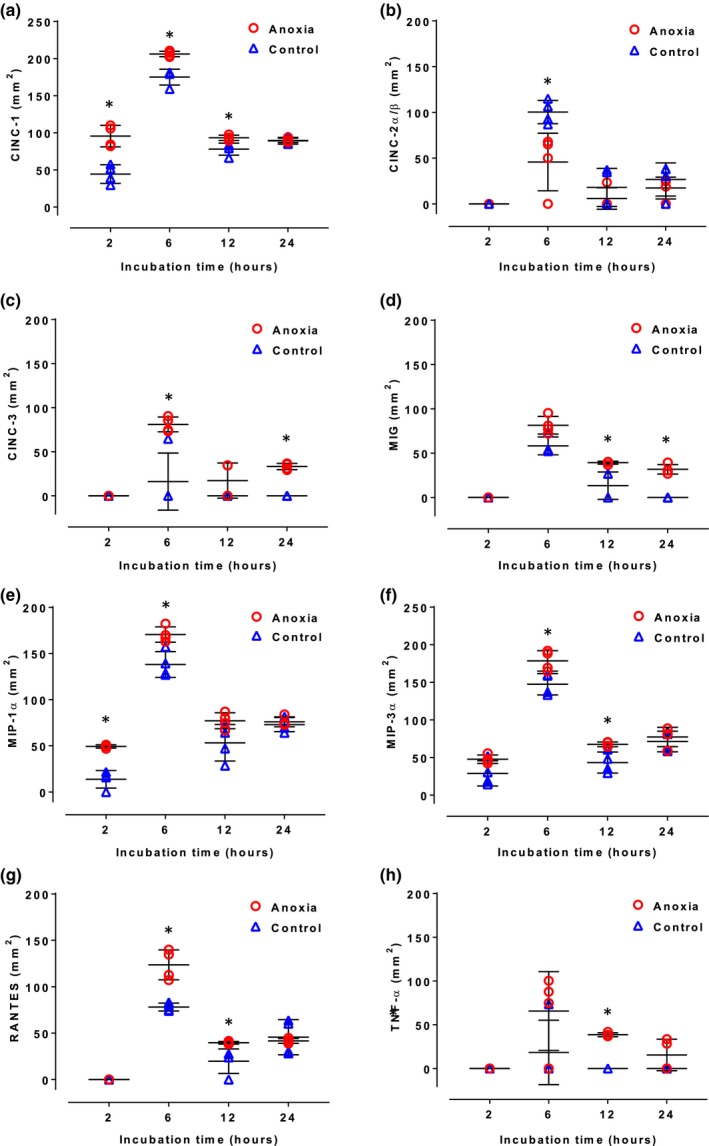
Secretion of CINC 1–3(A–C), MIG (D), MIP 1–2 (E–F), RANTES, and TNF‐α by astrocytes at 2, 6, 12, and 24 hr of incubation in control conditions (triangles) and subjected to anoxia (circles). Each symbol represents one sample. In some cases, data overlapped and hence it may appear that less than four samples are present for one time point. Long horizontal lines denote means, vertical lines denote standard deviations. Symbol * indicates significant difference between anoxia and control groups

Next, we explored secretion of several proinflammatory cytokines, including three cytokine‐induced neutrophil chemoattractants (CINCs 1–3), that are important in inducing recruitment and infiltration of neutrophils, and of MIG, MIP‐1α, MIP‐3α, RANTES, and TNF‐α. Concentrations of all these cytokines were higher in the cell culture media samples obtained after anoxia than in the corresponding control samples, except for CINC‐2, CINC‐3, MIG, RANTES, and TNFa after 2 hr anoxia (Figure 3). The difference in concentrations between samples taken after anoxia and the corresponding control samples was in some particular cases large and significant (e.g., CINC‐1 after 2 and 6 hr, CINC‐3 after 6 hr, MIG after 24 hr, RANTES after 6 hr and TNFa after 12 hr), but in all other cases it was only marginal and significant (Figure 3).

**Figure 4:**
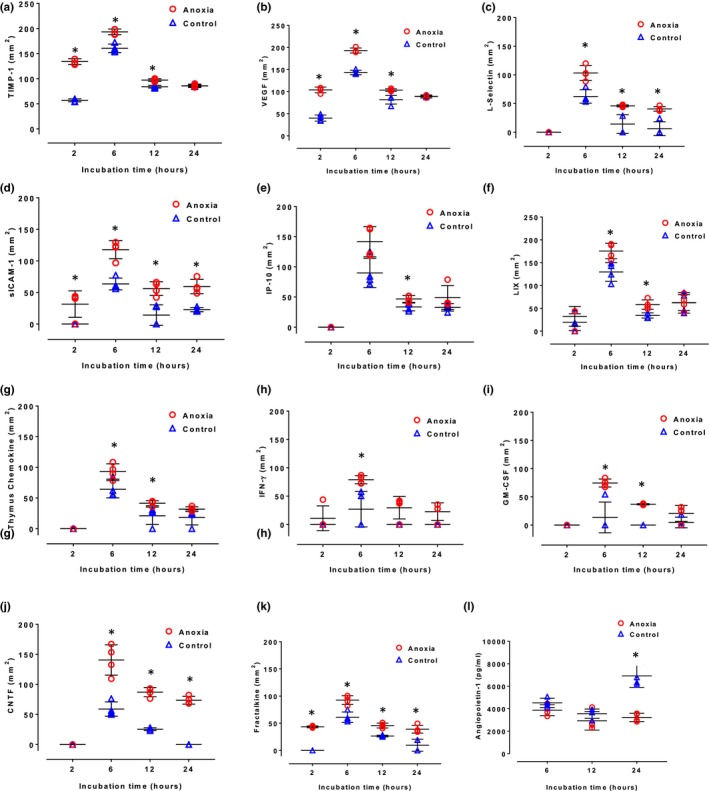
Secretion of TIMP (a), VEGF (b), l‐selectin (c), sICAM‐1 (d), IP‐10 (e), LIX (f), Thymus Chemokine (g), GM‐CSF (h), IFN‐γ (i), CNTF (j), Fractaline (k) by astrocytes at 2, 6, 12, and 24 hr and of Ang‐1 (l) at 6, 12, and 24 hr of incubation in control conditions (triangles) and subjected to anoxia (circles). Each symbol represents one sample. In some cases, data overlapped and hence it may appear that less than four samples were present for one‐time point. Long horizontal lines denote means, vertical lines denote standard deviations. Symbol * indicates significant difference between anoxia and control groups. VEGF, vascular endothelial growth factor

Next, we assessed concentrations of several cytokines that are involved in initiation/control of angiogenesis in the brain; VEGF (Jin, Mao, & Greenberg, 2000), TIMP‐1 (Cunningham, Wetzel, & Rosenberg, 2005), LIX, IP‐10 (Bajetto, Bonavia, Barbero, & Schettini, 2002), and angiopoietin‐1 (Hori, Ohtsuki, Hosoya, Nakashima, & Terasaki, 2004), as well as several cytokines/chemokines that control leukocyte attraction, extravasation and adhesion in the brain, Thymus chemokine (Imai et al., 1997), L‐selectin and sICAM‐1 (Lau & Yu, 2001), GM‐CSF, IFN‐γ (Choi, Lee, Lim, Satoh, & Kim, 2014; Lau & Yu, 2001), CNTF (Askvig & Watt, 2015), and fractalkine (Hatori, Nagai, Heisel, Ryu, & Kim, 2002). Again, no common pattern in changes in concentrations of these signaling molecules in cell culture media after anoxia could be established (Figure 4). Interestingly, concentrations of Ang‐1 were marginally lower after 6, 12 and substantially lower after 24 hr anoxia than in the corresponding controls. Concentrations of most other cytokines increased marginally after anoxia, while concentrations of TIMP‐1, VEGF, sICAM, and fractaline after 2 hr, VEGF, sICAM, and CNTF after 6 hr, GM‐CSF and CNTF after 12 hr and CNTF after 24 hr of anoxia increased substantially and significantly (Figure 4).

### 4.3 Molecular network analysis

**Figure 5:**
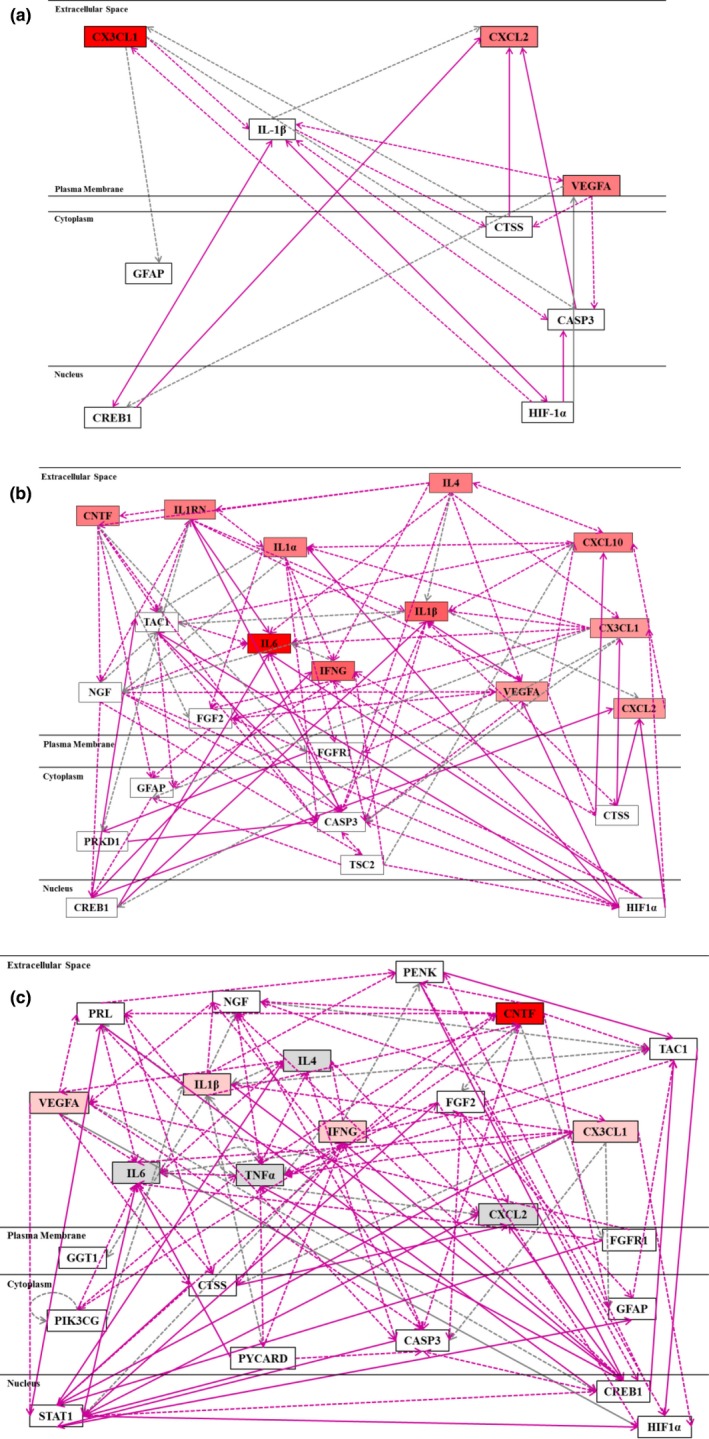
Molecular network analysis of signaling molecules/pathways upregulated in rat brain astrocytes during anoxia, based on cytokine and chemokine secretion after 2 hr (a), 6 hr (b), and 24 hr (c) anoxia. The IPA revealed the assignment and possible associations of changes in cytokines’ secretion to the following signaling pathways/molecules: hypoxia inducible factor 1 (HIF‐1α), interleukin 1 beta (IL‐1β), and cathepsin S (CTSS) after 2 hr; HIF‐1α, IL‐1β, ciliary neurotrophic factor (CNTF), and CTSS after 6 h and HIF‐1α, IL‐1β, CNTF, and CTSS after 24 hr

When the list of Entrez Gene IDs corresponding to 29 tested cytokines and chemokines secreted by astrocytes during anoxia were imported into the IPA, several molecular networks were identified. Changes in cytokine secretion at all time‐points tested were strongly associated to HIF‐1α signaling pathways, as well as IL‐1β and cathepsin S (CTSS) (Figure 5a–c). After 6 hr anoxia (Figure 5b) a more complex network was revealed than after 2 hr anoxia (Figure 5a); at this time point changes in cytokine secretion were also associated with pathways related to ciliary neurotrophic factor (CNTF). A similar network of pathways was revealed after 24 hr anoxia (Figure 5c) and the same four pathways/signaling molecules; HIF‐1α, IL‐1β, CTSS, and CNTF, appeared to be upregulated at this time point.

## 5 DISCUSSION

The main finding from this study is that anoxia triggers changes in secretion of cytokines and chemokines from rat astrocytes in primary culture in the absence of most other cells of the NVU and/or other injurious conditions. This could occur through HIF‐1α – mediated pathways. Changes in cytokines’ secretion in anoxic astrocytes were associated to upregulation of IL‐1b, a key cytokine that triggers inflammatory response, cathepsin S, a protease which plays a role in antigen presentation to immune cells and to CNTF, a potent neurotrophic/neuroprotective factor. However, as with any primary culture, these cultures could not be considered to be absolutely pure and a minor contamination with microglia could not be excluded, so the influence of microglia‐released cytokines and chemokines on astrocytes, which has been reported recently (Chen et al., 2016; Iizumi et al., 2016), although not very likely, could not be excluded.

Cytokines and chemokines constitute a group of structurally related molecules that play an important role in cell‐to‐cell communication in the CNS under both normal physiological conditions and in pathophysiological disturbances. Although there are many reports on effects of hypoxia or anoxia on expression and/or secretion of one, or a few, particular cytokines, this is the first study to explore the secretome profile of a large panel of these signaling molecules measured simultaneously under the same experimental conditions. In the present study, the cytokine and chemokine secretome of rat astrocytes subjected to anoxia indicates a molecular network that supports the view that these soluble factors are critically involved in regulation of cellular interactions and trafficking of immune cells.

A similar approach was used previously to study secretome profiles of cytokines and chemokines in human astrocytes that were stimulated by mediators of inflammation, with IL‐1β and TNF‐α (Choi et al., 2014). This study found that in control conditions, in the absence of stimulation, only G‐CSF, GM‐CSF, CXCL1, IL‐6, IL‐8, MCP‐1, MIF, and Serpin E1 could be detected in the cell culture medium. Following a 24 hr‐exposure to a mixture of IL‐1β and TNF‐α, the expression levels of six cytokines (G‐CSF, GM‐CSF, IL‐6, CXCL1, IL‐8, and Serpin E1) were increased substantially, while concentrations of MCP‐1 and MIF were reduced (Choi et al., 2014). In addition, activated astrocytes secreted several cytokines and chemokines that were not secreted by unstimulated astrocytes (IL‐1β, IL‐1ra, TNF‐α, IP‐10, MIP‐1α, RANTES, sICAM‐1) (Choi et al., 2014). Contrary with these findings, we found that rat astrocytes under control conditions secreted all 30 cytokines and chemokines that were explored, and that all cytokines, except Ang‐1, were upregulated (increased secretion) following anoxia, at least at some time points. The authors of the above mentioned study concluded that changes in expression of most of the cytokines and chemokines produced by nonstimulated and activated human astrocytes were mediated by the transcription factor NF‐kB, while IPA in this study of rodent astrocytes pointed to the HIF‐1α pathway; this difference may reflect the differences in the insults that challenged astrocytes in the two studies.

A key finding from our study is that anoxia, in the absence of any other challenge (e.g., stimulation by the mediators of inflammation or by hypoglycemia), and in the absence of other cell types present in the NVU, was sufficient to induce changes in secretion of a number of cytokines and chemokines. Hypoxia or anoxia causes a rapid upregulation of HIF‐1α pathway in most mammalian cells, and the role of this signaling pathway in cellular adaptation to hypoxia (Semenza, 2007), including neovascularization (Rey & Semenza, 2010), is well‐established and has been reviewed extensively. Our data are consistent with HIF‐1α pathway triggering cytokine expression in astrocytes but cannot exclude a possibility that the expression effects resulted from a different, HIF‐1α‐independent, mechanism. There are several lines of evidence showing that the HIF‐1 pathway could trigger expression and secretion of cytokines. This could be an important mechanism for activation of immune response in infection or trauma, since a reduction in the partial pressure of oxygen accompanies these conditions (Haddad & Harb, 2005). The expression of prolyl hydroxylases, which destabilize HIF‐1α through hydroxylation, were significantly decreased in MAPK phosphatase 1‐deficient bone marrow derived macrophages (BMDM) and exposure to LPS of these cells led to a large increase in IL‐1β production, which did not occur when HIF‐1α signaling was inhibited, indicating that a regulatory function for MAPK phosphatase 1 in modulating immune response is at least partially achieved through HIF‐1α pathway (Talwar et al., 2017). A cytokine‐mediated inflammation triggered by HIF‐2α has been revealed in reflux esophagitis (Souza, Bayeh, Spechler, Tambar, & Bruick, 2017). An in vitro study showed that in vitro infection with influenza H1N1 virus promotes the secretion of proinflammatory cytokines by inducing nuclear translocation of HIF‐1α (Guo et al., 2017). HIF‐1α appears to control expression of IL‐22 in CD4 T cells (Budda, Girton, Henderson, & Zenewicz, 2016). It is also important to stress that changes in secretion of cytokines and chemokines by astrocytes in this study were induced in the absence of any other cells of the NVU from the cultures; this finding is significant because it has been shown previously that tumor necrosis factor like weak inducer of apoptosis is secreted mainly by neurons during CI and binds to Fn14 on astrocytes, leading to proinflammatory molecule production (Saas et al., 2000).

The second main finding is that changes in the secretion of cytokines and chemokines during anoxia were strongly associated with signaling molecules that are related to the immune response or neuroprotection. Association of changes in cytokine secretion during anoxia to cathepsin S was intriguing. Cathepsin S is a lysosomal enzyme that belongs to the family of cysteine proteases; it promotes degradation of damaged or unwanted proteins, thus playing an important role in antigen presentation. It is expressed in antigen presenting cells, such as macrophages, B‐lymphocytes, and microglia (Wilkinson et al., 2015). A previous study has revealed that IFN‐gamma‐stimulated microglia possess active cathepsin L and cathepsin S (and thus can efficiently play a role in antigen presentation), while IFN‐gamma‐stimulated astrocytes expressed cathepsin L but not cathepsin S (Gresser, Weber, Hellwig, Riese, & Régnier‐Vigouroux, 2001). It has been believed that the lack of cathepsin S has a significant effect on the antigen‐presentation capacity of astrocytes. Our findings could imply that cathepsin S is upregulated in the brain by the signaling from anoxic astrocytes, which in turn implies that astrocytes play a role in antigen presentation during hypoxia/ischemia.

In addition to cathepsin S, our data also revealed association of the observed changes to IL‐1b after 6 and 24 hr anoxia. IL‐1b is a key mediator of the inflammatory response that is crucial for host‐defense responses to injury (Dinarello, 1996) by inducing upregulation of nuclear factor‐kappa B (NF‐kappaB) (Vykhovanets et al., 2009). This supports the hypothesis that hypoxic astrocytes play an important role in inducing inflammation in the brain.

An association of the observed changes to the signaling related to ciliary neurotrophic factor, was also revealed after 6 and 24 hr anoxia. This cytokine activates JAK1, JAK2, and TYK2 tyrosine kinases, which then phosphorylate STAT3. Phosphorylated STAT3 induces neurite outgrowth and neuronal migration (Pasquin, Sharma, & Gauchat, 2015) and suppresses AMP dependent kinase in some neurons (Steinberg et al., 2006), thereby promoting neuroprotective effects. Studies of the potential clinical application of this factor in patients suffering from Alzheimer disease, retinal degradation, ALS, and stroke have been undertaken, but did not reveal a real benefit for the patients (Chen & Wang, 2016; Kimura, Namekata, Guo, Harada, & Harada, 2016).

In conclusion, these findings collectively suggest that astrocytes challenged by anoxia play an important role in triggering immune response in the brain.

## CONFLICT OF INTEREST

Authors declare no conflict of interest.

## AUTHOR CONTRIBUTION

All authors had full access to the data in the study and take responsibility for the integrity of the data and the accuracy of the data analysis. Study concept and design: Z.R. J.C. Acquisition of data: Z. S. and L.A. Analysis and interpretation of the data: M.T., Z.R. Drafting of the manuscript: Z.S., Z.R. Critical revision of the manuscript for important intellectual content: M.T., J.C., Z.R. Statistical analysis: M.T. Obtained funding, administrative, technical and material support, study supervision: Z.R.

## DATA ACCESSIBILITY

All original data (two MS Excel files) are available at https://doi.org/10.6084/m9.figshare.5932738.

## Endnote

1 Authors used term “inflammation” to refer to inflammatory processes in the brain caused by cerebral ischemia and not neuroinflammation, since the latter term in fact refers to autoimmune inflammation in the NS (e.g., multiple sclerosis) (Filiou et al., 2014).

## References

[brb31013-bib-0001] Abbott, N. J. , Dolman, M. E. D. , Drndarski, S. , & Fredriksson, M. S. (2012). An improved in vitro blood–brain barrier model: Rat brain endothelial cells co‐cultured with astrocytes. Methods in Molecular Biology, 814, 415–430. 10.1007/978-1-61779-452-0 22144323

[brb31013-bib-0002] Akdis, M. , Aab, A. , Altunbulakli, C. , Azkur, K. , Costa, R. , Crameri, R. , … Akdis, C. A. (2016). Interleukins (from IL‐1 to IL‐38), interferons, transforming growth factor β, and TNF‐α: Receptors, functions, and roles in diseases. Journal of Allergy and Clinical Immunology, 138(4), 984–1010. 10.1016/j.jaci.2016.06.033 27577879

[brb31013-bib-0003] Askvig, J. , & Watt, J. (2015). The MAPK and PI3K pathways mediate CNTF‐induced neuronal survival and process outgrowth in hypothalamic organotypic cultures. Journal of Cell Communication and Signaling, 9(3), 217–231. 10.1007/s12079-015-0268-8 25698661 PMC4580676

[brb31013-bib-0004] Bajetto, A. , Bonavia, R. , Barbero, S. , & Schettini, G. (2002). Characterization of chemokines and their receptors in the central nervous system: Physiopathological implications. Journal of Neurochemistry, 82(6), 1311–1329. 10.1046/j.1471-4159.2002.01091.x 12354279

[brb31013-bib-0100] Barakat, R. , & Redzic, Z. (2015). Differential cytokine expression by brain microglia/macrophages in primary culture after oxygen glucose deprivation and their protective effects on astrocytes during anoxia. Fluids and Barriers of the CNS, 12, 6 10.1186/s12987-015-0002-1 25866619 PMC4392752

[brb31013-bib-0005] Budda, S. A. , Girton, A. , Henderson, J. G. , & Zenewicz, L. A. (2016). Transcription factor HIF‐1α controls expression of the cytokine IL‐22 in CD4 T cells. Journal of Immunology, 197(7), 2646–2652. 10.4049/jimmunol.1600250 27534553

[brb31013-bib-0101] Chen, J. H. , Tsai, C. , Lin, H. Y. , Huang, C. F. , Leung, Y. M. , Lai, S. W. , … Lin, C. (2016). Interlukin‐18 Is a Pivot Regulatory Factor on Matrix Metalloproteinase‐13 Expression and Brain Astrocytic Migration. Molecular Neurobiology, 53(9), 6218–6227. 10.1007/s12035-015-9529-z 26558633

[brb31013-bib-0006] Chen, X. , & Wang, K. (2016). The fate of medications evaluated for ischemic stroke pharmacotherapy over the period 1995–2015. Acta Pharmaceutica Sinica B, 6(6), 522–530. 10.1016/j.apsb.2016.06.013 27818918 PMC5071630

[brb31013-bib-0007] Choi, S. S. , Lee, H. J. , Lim, I. , Satoh, J. , & Kim, S. U. (2014). Human astrocytes: Secretome profiles of cytokines and chemokines. PLoS ONE, 9(4), e92325 10.1371/journal.pone.0092325 24691121 PMC3972155

[brb31013-bib-0008] Cunningham, L. , Wetzel, M. , & Rosenberg, G. (2005). Multiple roles for MMPs and TIMPs in cerebral ischemia. Glia, 50(4), 329–339. 10.1002/(ISSN)1098-1136 15846802

[brb31013-bib-0009] Dinarello, C. A. (1996). Biologic basis for interleukin‐1 in disease. Blood, 87(6), 2095–2147. 8630372

[brb31013-bib-0010] Dirnagl, U. (2012). Pathobiology of injury after stroke: The neurovascular unit and beyond. Annals of the New York Academy of Sciences, 1268, 21 10.1111/j.1749-6632.2012.06691.x 22994217

[brb31013-bib-0011] Farina, C. , Aloisi, F. , & Meinl, E. (2007). Astrocytes are active players in cerebral innate immunity. Trends in Immunology, 28(3), 138–145. 10.1016/j.it.2007.01.005 17276138

[brb31013-bib-0012] Filiou, M. D. , Arefin, A. S. , Moscato, P. , & Graeber, M. B. (2014). ‘Neuroinflammation’ differs categorically from inflammation: Transcriptomes of Alzheimer’s disease, Parkinson’s disease, schizophrenia and inflammatory diseases compared. Neurogenetics, 15(3), 201–212. 10.1007/s10048-014-0409-x 24928144

[brb31013-bib-0013] Gresser, O. , Weber, E. , Hellwig, A. , Riese, S. , & Régnier‐Vigouroux, A. (2001). Immunocompetent astrocytes and microglia display major differences in the processing of the invariant chain and in the expression of active cathepsin L and cathepsin S. European Journal of Immunology, 31(6), 1813–1824. 10.1002/(ISSN)1521-4141 11433378

[brb31013-bib-0014] Guo, X. , Zhu, Z. , Zhang, W. , Meng, X. , Zhu, Y. , Han, P. , … Wang, R. (2017). Nuclear translocation of HIF‐1α induced by influenza A (H1N1) infection is critical to the production of proinflammatory cytokines. Emerging Microbes & Infections, 6(5), e39 10.1038/emi.2017.21 28536432 PMC5520484

[brb31013-bib-0015] Haddad, J. J. , & Harb, H. L. (2005). Cytokines and the regulation of hypoxia‐inducible factor (HIF)‐1alpha. International Immunopharmacology, 5(3), 461–483. 10.1016/j.intimp.2004.11.009 15683844

[brb31013-bib-0016] Hatori, K. , Nagai, A. , Heisel, R. , Ryu, J. , & Kim, S. (2002). Fractalkine and fractalkine receptors in human neurons and glial cells. Journal of Neuroscience Research, 69(3), 418–426. 10.1002/(ISSN)1097-4547 12125082

[brb31013-bib-0017] Hori, S. , Ohtsuki, S. , Hosoya, K. , Nakashima, E. , & Terasaki, T. (2004). A pericyte‐derived angiopoietin‐1 multimeric complex induces occludin gene expression in brain capillary endothelial cells through Tie‐2 activation in vitro. Journal of Neurochemistry, 89, 503–513. 10.1111/j.1471-4159.2004.02343.x 15056293

[brb31013-bib-0018] Hu, J. , Saito, T. , Abe, K. , & Deguchi, T. (1997). Increase of ciliary neurotrophic factor (CNTF) in the ischemic rat brain as determined by a sensitive enzyme‐linked immunoassay. Neurological Research, 19(6), 593–598. 10.1080/01616412.1997.11740865 9427958

[brb31013-bib-0019] Iadecola, C. (2017). The neurovascular unit coming of age: A journey through neurovascular coupling in health and disease. Neuron, 96(1), 17–42. 10.1016/j.neuron.2017.07.030 28957666 PMC5657612

[brb31013-bib-0020] Igarashi, Y. , Utsumi, H. , Chiba, H. , Yamada‐Sasamori, Y. , Tobioka, H. , Kamimura, Y. , … Sawada, N. (1999). Glial cell line‐derived neurotrophic factor induces barrier function of endothelial cells forming the blood–brain barrier. Biochemical and Biophysical Research Communications, 261(1), 108–112. 10.1006/bbrc.1999.0992 10405331

[brb31013-bib-0021] Iizumi, T. , Takahashi, S. , Mashima, K. , Minami, K. , Izawa, Y. , Abe, T. , … Suzuki, N. (2016). A possible role of microglia‐derived nitric oxide by lipopolysaccharide in activation of astroglial pentose‐phosphate pathway via the Keap1/Nrf2 system. Journal of Neuroinflammation, 13(1), 99 10.1186/s12974-016-0564-0 27143001 PMC4855896

[brb31013-bib-0022] Imai, T. , Baba, M. , Nishimura, M. , Kakizaki, M. , Takagi, S. , & Yoshie, O. (1997). The T cell‐directed CC chemokine TARC is a highly specific biological ligand for CC chemokine receptor 4. Journal of Biological Chemistry, 272(23), 15036–15042. 10.1074/jbc.272.23.15036 9169480

[brb31013-bib-0023] Jin, K. L. , Mao, X. O. , & Greenberg, D. A. (2000). Vascular endothelial growth factor: Direct neuroprotective effect in in vitro ischemia. Proceedings of the National Academy of Sciences USA, 97, 10242–10247. 10.1073/pnas.97.18.10242 PMC27841 10963684

[brb31013-bib-0024] Jones, K. A. , Maltby, S. , Plank, M. W. , Kluge, M. , Nilsson, M. , Foster, P. S. , & Walker, F. R. (2018). Peripheral immune cells infiltrate into sites of secondary neurodegeneration after ischemic stroke. Brain, Behaviour and Immunity, 67, 299–307. 10.1016/j.bbi.2017.09.006 28911981

[brb31013-bib-0025] Kim, J. H. , Min, K. J. , Seol, W. , Jou, I. , & Joe, E. H. (2010). Astrocytes in injury states rapidly produce anti‐inflammatory factors and attenuate microglial inflammatory responses. Journal of Neurochemistry, 115(5), 1161–1171. 10.1111/j.1471-4159.2010.07004.x 21039520

[brb31013-bib-0026] Kimura, A. , Namekata, K. , Guo, X. , Harada, C. , & Harada, T. (2016). Neuroprotection, growth factors and BDNF‐TrkB signalling in retinal degeneration. International Journal of Molecular Sciences, 17(9), 1584 10.3390/ijms17091584 PMC5037849 27657046

[brb31013-bib-0027] Lau, L. , & Yu, A. (2001). Astrocytes produce and release interleukin‐1, interleukin‐6, tumor necrosis factor alpha and interferon‐gamma following traumatic and metabolic injury. Journal of Neurotrauma, 18(3), 351–359. 10.1089/08977150151071035 11284554

[brb31013-bib-0028] Lu, W. , Maheshwari, A. , Misiuta, I. , Fox, S. , Chen, N. , Zigova, T. , … Calhoun, D. A. (2005). Neutrophil‐specific chemokines are produced by astrocytic cells but not by neuronal cells. Developmental Brain Research, 155(2), 127–134. 10.1016/j.devbrainres.2005.01.004 15804401

[brb31013-bib-0029] Pasquin, S. , Sharma, M. , & Gauchat, J. F. (2015). Ciliary neurotrophic factor (CNTF): New facets of an old molecule for treating neurodegenerative and metabolic syndrome pathologies. Cytokine Growth Factor Reviews, 26(5), 507–515. 10.1016/j.cytogfr.2015.07.007 26187860

[brb31013-bib-0030] Rey, S. , & Semenza, G. L. (2010). Hypoxia‐inducible factor‐1‐dependent mechanisms of vascularization and vascular remodelling. Cardiovascular Research, 86(2), 236–242. 10.1093/cvr/cvq045 20164116 PMC2856192

[brb31013-bib-0031] Saas, P. , Boucraut, J. , Walker, P. R. , Quiquerez, A. L. , Billot, M. , Desplat‐Jego, S. , … Dietrich, P. Y. (2000). TWEAK stimulation of astrocytes and the proinflammatory consequences. Glia, 32(1), 102–107. 10.1002/(ISSN)1098-1136 10975915

[brb31013-bib-0032] Semenza, G. L. (2007). Life with oxygen. Science, 318(5847), 62–64. 10.1126/science.1147949 17916722

[brb31013-bib-0033] Siniscalchi, A. , Gallelli, L. , Malferrari, G. , Pirritano, D. , Serra, R. , Santangelo, E. , & De Sarro, G. (2014). Cerebral stroke injury: The role of cytokines and brain inflammation. Journal of Basic and Clinical Physiology and Pharmacology, 25(2), 131–137. 10.1515/jbcpp-2013-0121 24515999

[brb31013-bib-0034] Souza, R. F. , Bayeh, L. , Spechler, S. J. , Tambar, U. K. , & Bruick, R. K. (2017). A new paradigm for GERD pathogenesis. Not acid injury, but cytokine‐mediated inflammation driven by HIF‐2α: A potential role for targeting HIF‐2α to prevent and treat reflux esophagitis. Current Opinion in Pharmacology, 37, 93–99. 10.1016/j.coph.2017.10.004 29112883 PMC5922421

[brb31013-bib-0035] Steinberg, G. R. , Watt, M. J. , Fam, B. C. , Proietto, J. , Andrikopoulos, S. , Allen, A. M. , … Kemp, B. E. (2006). Ciliary neurotrophic factor suppresses hypothalamic AMP‐kinase signaling in leptin‐resistant obese mice. Endocrinology, 147(8), 3906–3914. 10.1210/en.2005-1587 16675525

[brb31013-bib-0036] Sweeney, M. D. , Ayyadurai, S. , & Zlokovic, B. (2016). Pericytes of the neurovascular unit: Key functions and signaling pathways. Nature Neuroscience, 19(6), 771–783. 10.1038/nn.4288 27227366 PMC5745011

[brb31013-bib-0037] Talwar, H. , Bauerfeld, C. , Bouhamdan, M. , Farshi, P. , Liu, Y. , & Samavati, L. (2017). MKP‐1 negatively regulates LPS‐mediated IL‐1β production through p38 activation and HIF‐1α expression. Cellular Signalling, 34, 1–10. 10.1016/j.cellsig.2017.02.018 28238855 PMC5410178

[brb31013-bib-0038] Tuttolomondo, A. , Di Raimondo, D. , di Sciacca, R. , Pinto, A. , & Licata, G. (2008). Inflammatory cytokines in acute ischemic stroke. Current Pharmaceutical Design, 14(33), 3574–3589. 10.2174/138161208786848739 19075734

[brb31013-bib-0039] Vykhovanets, E. V. , Shukla, S. , MacLennan, G. T. , Vykhovanets, O. V. , Bodner, D. R. , & Gupta, S. (2009). Il‐1 beta‐induced post‐transition effect of NF‐kappaB provides time‐dependent wave of signals for initial phase of intrapostatic inflammation. Prostate, 69(6), 633–643. 10.1002/pros.20916 19170127 PMC2669895

[brb31013-bib-0103] Wilkinson, R. D. , Williams, R. , Scott, C. J. , & Burden, R. E. (2015). Cathepsin S: therapeutic, diagnostic, and prognostic potential. Biological Chemistry, 396(8), 867–882. 10.1515/hsz-2015-0114 25872877

