# Supplementary figures and images for: Rat astrocytes during anoxia: Secretome profile of cytokines and chemokines
Source: Brain Behav. 2018 Jun 4;8(7):e01013. doi: 10.1002/brb3.1013 (PMC6043693; doi:10.1002/brb3.1013)

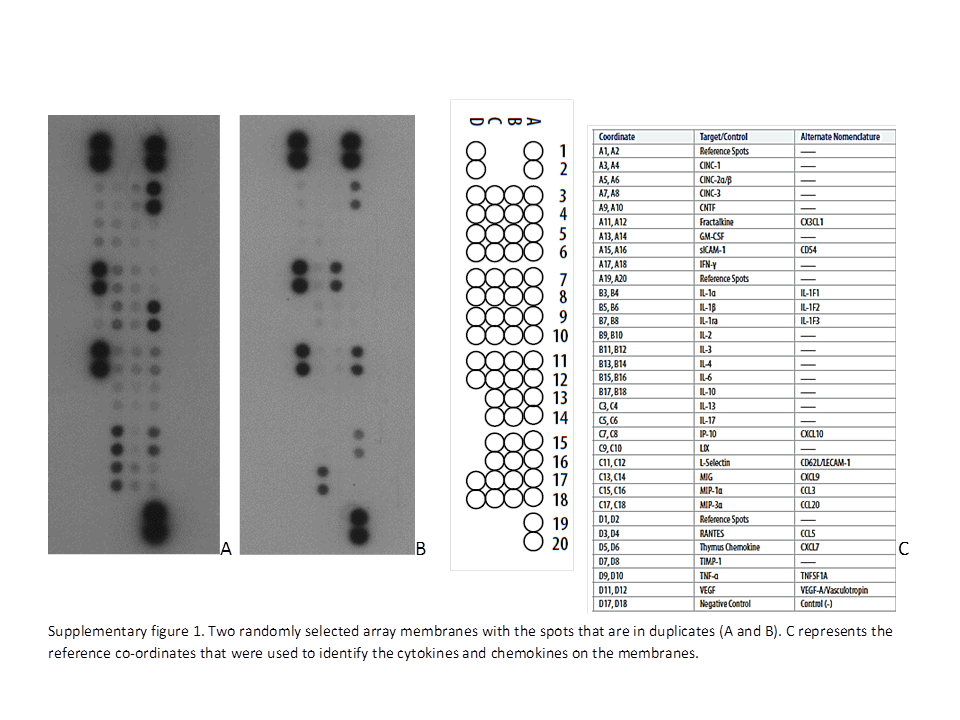

Supplement: Supplementary file 1 [file BRB3-8-e01013-s001.tif]
